# A Precise and Scalable Indoor Positioning System Using Cross-Modal Knowledge Distillation

**DOI:** 10.3390/s24227322

**Published:** 2024-11-16

**Authors:** Hamada Rizk, Ahmed Elmogy, Mohamed Rihan, Hirozumi Yamaguchi

**Affiliations:** 1 Computers & Control Engineering Department, Tanta University, Tanta 31527, Egypt; hamada_rizk@f-eng.tanta.edu.eg; 2 Graduate School of Information Science and Technology, Osaka University, Osaka 565-0871, Japan; h-yamagu@ist.osaka-u.ac.jp; 3 Faculty of Computer Engineering & Sciences, Prince Sattam Bin Abdelaziz University, Al-Kharj 16278, Saudi Arabia; 4 Faculty of Engineering, Tanta University, Tanta 31527, Egypt; 5 Department of Communications Engineering, University of Bremen, 28725 Bremen, Germany; elmeligy@ant.uni-bremen.de

**Keywords:** indoor localization, deep learning, fingerprinting, round trip time, knowledge distillation

## Abstract

User location has emerged as a pivotal factor in human-centered environments, driving applications like tracking, navigation, healthcare, and emergency response that align with Sustainable Development Goals (SDGs). However, accurate indoor localization remains challenging due to the limitations of GPS in indoor settings, where signal interference and reflections disrupt satellite connections. While Received Signal Strength Indicator (RSSI) methods are commonly employed, they are affected by environmental noise, multipath fading, and signal interference. Round-Trip Time (RTT)-based localization techniques provide a more resilient alternative but are not universally supported across access points due to infrastructure limitations. To address these challenges, we introduce *DistilLoc*: a cross-knowledge distillation framework that transfers knowledge from an RTT-based teacher model to an RSSI-based student model. By applying a teacher–student architecture, where the RTT model (teacher) trains the RSSI model (student), *DistilLoc* enhances RSSI-based localization with the accuracy and robustness of RTT without requiring RTT data during deployment. At the core of *DistilLoc*, the FNet architecture is employed for its computational efficiency and capacity to capture complex relationships among RSSI signals from multiple access points. This enables the student model to learn a robust mapping from RSSI measurements to precise location estimates, reducing computational demands while improving scalability. Evaluation in two cluttered indoor environments of varying sizes using Android devices and Google WiFi access points, *DistilLoc* achieved sub-meter localization accuracy, with median errors of 0.42 m and 0.32 m, respectively, demonstrating improvements of 267% over conventional RSSI methods and 496% over multilateration-based approaches. These results validate *DistilLoc* as a scalable, accurate solution for indoor localization, enabling intelligent, resource-efficient urban environments that contribute to SDG 9 (Industry, Innovation, and Infrastructure) and SDG 11 (Sustainable Cities and Communities).

## 1. Introduction

User location has emerged as a key factor in human-centered environments, greatly enhancing applications and services such as tracking, navigation, healthcare, and emergency response [1,2,3]. For instance, improving localization accuracy to cut emergency response times by even one minute could save over 10,000 lives each year in the United States [4]. Since people spend the majority of their time indoors, considerable attention has been focused on indoor localization. Although GPS is the standard for outdoor localization, it is ineffective indoors due to high levels of signal interference and reflections [5], which disrupt the line-of-sight to satellites. Consequently, indoor localization has become a focal point for research aiming to find accurate and ubiquitous alternatives to GPS in indoor settings [6,7,8,9,10,11]. Various technologies have been explored for this purpose, including WiFi, Radio Frequency Identification (RFID), Bluetooth, Ultra-Wideband (UWB), cellular, Zigbee, and Inertial Measurement Units (IMU) [12,13,14,15,16]. Each technology has unique advantages that make it suitable for specific applications. WiFi has become widely adopted, largely due to its extensive coverage and the IEEE 802.11 standard’s support across most mobile devices [8,17,18,19,20,21,22,23,24,25].

Various techniques have been proposed to tackle indoor localization challenges, including multilateration, fingerprinting, angle of arrival, and time-based methods [26,27]. Of these, fingerprinting and time-based techniques are the most widely studied. Fingerprinting [28,29,30] is particularly popular for its strong performance, especially when enhanced with deep learning. This approach involves creating a fingerprint database, which stores WiFi signal signatures collected at specific reference points throughout the area of interest. The database is then used to develop a model for estimating user location based on received signals during runtime. The models used in fingerprinting can be deterministic [31], probabilistic [32], or based on machine learning [33]. Probabilistic models that generally handle the inherent noise in wireless signals are better than deterministic models [27]. However, they often assume in-dependency among access points (APs) to avoid the curse of dimensionality [34], which can lead to information loss [27]. Deep learning has been widely employed to model the joint distribution of signals received from APs, resulting in enhanced localization accuracy. However, fingerprinting methods frequently encounter challenges due to RSSI fluctuations and signal interference from obstacles, multipath fading, indoor noise, and hardware variability.

To tackle these challenges, time-based techniques have been explored. These methods calculate the distance between a mobile device (e.g., a smartphone) and access points (APs) by measuring the signal’s propagation time and knowing the speed at which the signal travels. Some common approaches include Time of Arrival (ToA) [35,36], Time Difference of Arrival (TDoA) [37], and RTT (Round-Trip Time) [38,39]. ToA and TDoA require precise time synchronization between all devices. In contrast, RTT measures the time for a signal to travel to a target node and return, requiring only one clock, thus reducing synchronization issues.

Unlike RSSI-based methods, RTT is more resilient to the challenges posed by cluttered indoor environments. RTT is less affected by multipath interference, where signals bounce off surfaces and cause multiple signal paths. This interference can severely distort RSSI measurements but has a diminished effect on RTT due to its ability to distinguish between the direct signal path and reflected paths based on time delays. Additionally, RTT is less vulnerable to signal attenuation, which occurs when signals weaken as they pass through walls or obstacles. Even with reduced signal strength, RTT can maintain accurate time measurements. Furthermore, RTT is more robust against variations in transmission power and radio interference, as these factors do not significantly influence the time it takes for the signal to travel between the device and the access point. The Fine Time Measurement (FTM) protocol introduced by the IEEE 802.11-2016 standard facilitates the measurement of RTT between mobile phones and APs. This protocol’s adoption is growing among commercial APs and consumer mobile phones, making time-based techniques increasingly viable for practical indoor localization solutions.

Despite these advantages, the adoption of RTT-based localization solutions is limited by the fact that not all access points and devices are RTT-capable. In contrast, most devices can measure RSSI. This disparity restricts the widespread implementation of RTT-based solutions, as relying solely on RTT would necessitate significant infrastructure upgrades, which can be costly and impractical. To address this limitation, we present *DistilLoc*: a cross-knowledge distillation framework that leverages machine learning to transfer knowledge from an RTT-based teacher model to an RSSI-based student model. Our approach involves constructing a fingerprinting database where both RSSI and RTT measurements are collected during the offline phase at various reference points within the area of interest. These fingerprints serve as the training dataset for the teacher and student models. By using the RTT model as the teacher, which offers more accurate distance measurements and is more resilient to environmental challenges such as multipath interference, signal attenuation, transmission power variations, and radio interference, the RSSI model is trained to emulate the RTT measurements. This teacher–student framework allows the RSSI model to inherit the robustness of RTT while maintaining lower computational demands, particularly in environments where RTT data are unavailable during runtime.

During the training phase, the RTT model (teacher) is used to generate high-precision localization predictions, which are then used as the ground truth for training the RSSI model (student). The F-Net architecture is employed to effectively capture the complex relationships and dependencies between the RSSI signals from different access points, allowing the student model to learn a robust mapping from RSSI measurements to accurate location estimates. This cross-knowledge distillation approach enables the seamless integration of heterogeneous network data, creating a unified localization system that operates effectively irrespective of the specific capabilities of individual access points or devices. Given that RTT-capable devices and access points can be cost-prohibitive and less prevalent, relying exclusively on RTT is not feasible. The proposed *DistilLoc* system, therefore, offers a practical solution by enhancing the performance of existing RSSI-based systems without necessitating a complete overhaul of the existing infrastructure. This approach not only improves localization accuracy but also ensures scalability and cost-effectiveness in diverse indoor environments. By using RTT as the teacher model, which generally provides more precise distance measurements, the RSSI model can be trained to mimic the more accurate RTT measurements. This process can significantly improve the accuracy of the RSSI-based localization, especially in environments where RTT is not available at all points. The approach facilitates the seamless integration of heterogeneous networks, enabling a unified localization system that can operate effectively regardless of the capabilities of individual access points or devices. Since RTT-capable devices and access points can be more expensive and less widespread, relying solely on RTT could be impractical. Cross-knowledge distillation allows for improved performance without the need for a complete overhaul of the existing infrastructure.

By enabling accurate and real-time indoor positioning on resource-constrained devices, DistilLoc provides a practical solution that can be deployed in diverse environments, from smart factories to smart homes, fostering innovation in industries that rely on precise indoor navigation and improving the infrastructure that supports these technologies. Thus, the proposed approach makes a significant contribution to the achievement of Sustainable Development Goal (SDG) 9: Industry, Innovation, and Infrastructure. Additionally, this work supports Sustainable Development Goal (SDG) 11: Sustainable Cities and Communities, by enabling the creation of intelligent, resource-efficient urban environments. As cities increasingly embrace smart technologies, accurate and scalable localization solutions are essential for enhancing urban mobility, improving safety, and enabling better management of public spaces. DistilLoc’s ability to achieve high accuracy across heterogeneous devices and diverse indoor environments makes it a key enabler for applications like smart buildings, indoor navigation for the visually impaired, and more efficient use of urban infrastructure, contributing to more sustainable and livable cities.

We implemented and evaluated *DistilLoc* using different Android phones in two different cluttered environments. A large environment 629 m^2^ in area and a small one of 141 m^2^ was used. Seven commercial Google WiFi access points (APs) are deployed in each environment, alongside traditional non-RTT-enabled APs, which can serve as additional sources of interference. The obtained results show that *DistilLoc* achieves a sub-meter localization accuracy for both indoor environments with a median localization error of 0.42 m and 0.32 m for the two environments, respectively. These results demonstrate an improvement of at least 267% in accuracy over traditional RSSI fingerprinting methods, and an enhancement of at least 496% compared to the accuracy of the ranging-based multilateration localization approach. This level of accuracy is sustained across heterogeneous devices, establishing *DistilLoc* as a robust and precise indoor localization technique.

The developed *DistilLoc* approach is novel compared to existing methods in several ways:

- **Cross-Modal Distillation with Softened Outputs:** The use of temperature-scaled softmax outputs for distillation is specifically tailored for cross-modal scenarios, allowing the student model to benefit from the richer feature set of the RTT model without requiring access to RTT data during deployment. A novel data-driven fusion and feature learning method has been developed to extract a correlated representation of RTT and RSSI.
- **Integration of F-Net:** Unlike traditional localization methods that rely on simple feature extraction techniques, our use of F-Net enables the efficient handling of high-dimensional signal data, providing a more scalable solution for real-time applications.
- **Scalability and Practicality:** By focusing on enhancing RSSI-based models, the proposed approach does not require significant infrastructure changes, making it highly practical for deployment in existing networks. This is in contrast to other methods that either require dedicated hardware for RTT measurements or complex fusion strategies that are not easily scalable.
- **Empirical Validation:** The proposed system has been rigorously tested in two indoor environments, demonstrating its robustness and superior performance compared to the state-of-the-art localization systems.

The remainder of this paper is organized as follows. Section 2 offers a brief introduction to RTT and knowledge distillation. Section 3 reviews the most pertinent work related to the proposed system. In Section 4, we provide a comprehensive overview of the *DistilLoc* system architecture and its various components. The proposed localization system is detailed in Section 5. Section 6 assesses the different parameters of *DistilLoc* and compares its overall performance to other approaches. Finally, Section 7 wraps up the paper and discusses potential future work.

## 2. Background

### 2.1. Round Trip Time (RTT)

RTT is a time-based technique primarily used for determining distance. In this study, it will be employed to measure the distance between two WiFi stations: the user’s mobile device and the access point. A key advantage of the RTT technique is that it can measure the distance between two stations without requiring explicit synchronization, which is a significant challenge in time-based localization.

RTT works by measuring the time it takes for a signal to travel from one device to another and back. In the context of WiFi, a signal is sent from a mobile device to AP. The AP immediately sends a response back to the mobile device. By calculating the time taken for this round trip and knowing the speed of the signal, the distance between the two devices can be estimated. This process does not require precise synchronization between the devices, making it an effective technique for distance measurement in wireless networks. Another advantage of using the RTT technique is its capability to calculate distance on the edge, thereby ensuring user privacy.

**Figure 1:**
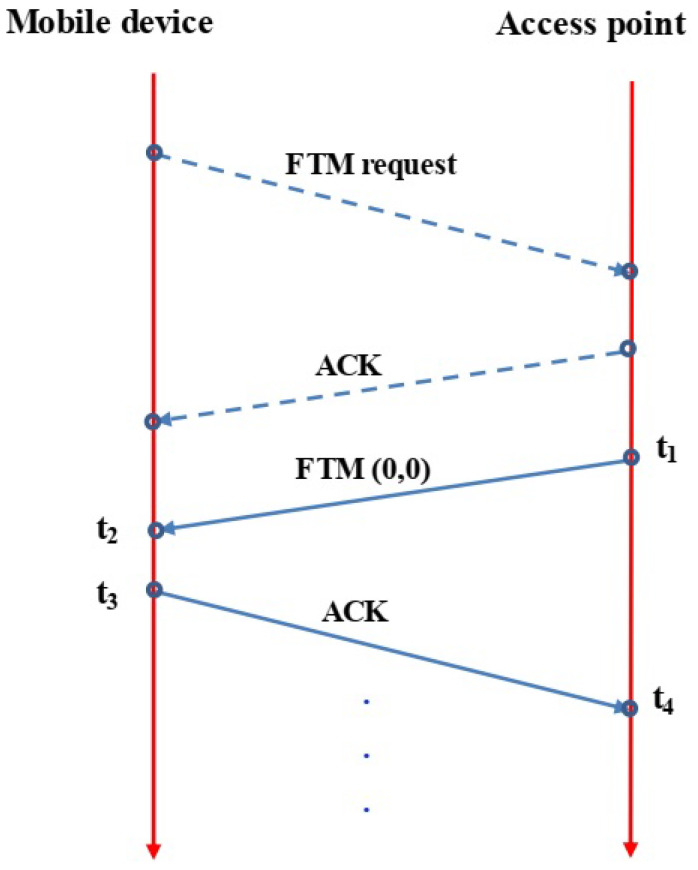
FTM protocol.

As depicted in Figure 1, the process starts with the mobile device sending an FTM request to the access point to check its availability. Upon receiving the request, the access point responds with an ACK signal if it is available. Following this, the mobile device can calculate the round trip time by transmitting multiple FTM packets. The mobile device processing time (TP) is computed by Equation (1).

(1) $$T_{p}=t_{3}-t_{2}\;$$

The round trip time (*RTT*) is calculated as in Equation (2).

(2) $$RTT=t_{4}-t_{1}-T_{p}\;$$

Equation (3) shows how to compute the distance (*D*) between the mobile device and the access point.

(3) $$D=\frac{1}{2}RTT\times C\;$$

The light speed $C=3\times 10^{8}$ m/s.

In contrast to multi-lateration approaches [20,40,41], *DistilLoc* employs the collected RTT values (acquired through the FTM protocol) as fingerprints, providing unique signatures for each location. This will be outlined in detail in the following section.

### 2.2. Knowledge Distillation

Knowledge distillation (KD) is a powerful technique for transferring knowledge from a larger, more complex model (the teacher) to a smaller, more efficient model (the student). The goal is to retain the performance benefits of the larger model while reducing the computational complexity, enabling deployment in resource-constrained environments.

In the knowledge distillation framework, let $x\in R^{d}$ denote the input data, and let y∈{1,2,…,C} represent the corresponding true labels, where *C* is the number of classes. The teacher model is a pre-trained model with parameters $\theta_{T}$, and it outputs a set of logits $z_{T}=f_{T}\left(x;\theta_{T}\right)$. These logits are then passed through a softmax function to obtain the predicted probability distribution:

(4) $$p_{T}=\mathrm{softmax}\left(z_{T}\right)=\left[\frac{\exp(z_{T,1})}{\sum_{j=1}^{C}\exp\left(z_{T,j}\right)},\ldots ,\frac{\exp(z_{T,C})}{\sum_{j=1}^{C}\exp\left(z_{T,j}\right)}\right]$$

Similarly, the student model, with parameters $\theta_{S}$, produces its own set of logits $z_{S}=f_{S}\left(x;\theta_{S}\right)$ and corresponding probability distribution:

(5) $$p_{S}=\mathrm{softmax}\left(z_{S}\right)=\left[\frac{\exp(z_{S,1})}{\sum_{j=1}^{C}\exp\left(z_{S,j}\right)},\ldots ,\frac{\exp(z_{S,C})}{\sum_{j=1}^{C}\exp\left(z_{S,j}\right)}\right]$$

The central idea in knowledge distillation is to train the student model such that its output distribution $p_{S}$ closely matches the teacher’s output distribution $p_{T}$, while also ensuring that the student performs well on the actual classification task.

A key component of knowledge distillation is the use of softened probability distributions, achieved by introducing a temperature parameter T>1 in the softmax function. The logits from both the teacher and the student are softened as follows:

(6) $$p_{T}^{\left(T\right)}=\mathrm{softmax}\left(\frac{z_{T}}{T}\right)=\left[\frac{\exp(z_{T,1}/T)}{\sum_{j=1}^{C}\exp\left(z_{T,j}/T\right)},\ldots ,\frac{\exp(z_{T,C}/T)}{\sum_{j=1}^{C}\exp\left(z_{T,j}/T\right)}\right]$$

(7) $$p_{S}^{\left(T\right)}=\mathrm{softmax}\left(\frac{z_{S}}{T}\right)=\left[\frac{\exp(z_{S,1}/T)}{\sum_{j=1}^{C}\exp\left(z_{S,j}/T\right)},\ldots ,\frac{\exp(z_{S,C}/T)}{\sum_{j=1}^{C}\exp\left(z_{S,j}/T\right)}\right]$$

The temperature *T* controls the softness of the output distribution. When T=1, the distribution is the same as the original softmax output. When T>1, the distribution becomes softer, spreading the probabilities more evenly across the classes. This softened distribution contains richer information about the inter-class relationships, which the student model can learn from.

The loss function used to train the student model is a weighted combination of two components: the supervised loss and the distillation loss. The supervised cross-entropy loss ensures that the student model performs well on the actual classification task by minimizing the cross-entropy between the true labels, denoted as y, and the student’s predictions, $p_{S}$. This loss is calculated as follows:

(8) $$L_{\mathrm{CE}}=-\sum_{i=1}^{C}y_{i}\log\left(p_{S,i}\right),$$

where $y_{i}$ represents the one-hot encoded true label. In addition to this, the distillation loss encourages the student model to mimic the softened output distribution of the teacher model. This loss is typically defined using the Kullback–Leibler (KL) divergence between the softened distributions of the teacher and the student, formulated as follows:

(9) $$L_{\mathrm{KD}}=T^{2}\cdot \mathrm{KL}\left(p_{T}^{\left(T\right)}∥p_{S}^{\left(T\right)}\right)=T^{2}\sum_{i=1}^{C}p_{T,i}^{\left(T\right)}\log\left(\frac{p_{T,i}^{\left(T\right)}}{p_{S,i}^{\left(T\right)}}\right).$$

The KL divergence measures how one probability distribution diverges from another reference distribution. The factor $T^{2}$ is introduced to adjust the gradients during backpropagation, ensuring that the distillation loss is appropriately scaled despite the temperature’s effect.

The total loss function used to train the student model is as follows:

(10) $$L=\left(1-\alpha \right)\cdot L_{\mathrm{CE}}+\alpha \cdot L_{\mathrm{KD}}$$

where α is a hyperparameter that balances the importance of the two losses.

Knowledge distillation is effective for several reasons. First, the softened labels from the teacher model provide richer information than hard labels, as they include not only the correct class but also the relative probabilities of incorrect classes. This enables the student model to learn smoother decision boundaries, which can generalize better to new data. Additionally, training on the softened outputs of the teacher model reduces the likelihood of overfitting, as these outputs are generally more generalized compared to hard labels, particularly when the teacher is a high-capacity model. Furthermore, the student model benefits from learning from a stronger, more powerful teacher model that has captured complex patterns in the data. By mimicking the teacher, the student inherits some of these learned patterns, leading to improved performance. Finally, the softened probabilities create a smoother optimization landscape, resulting in more stable and efficient optimization during backpropagation, which is especially advantageous when training deep neural networks are used.

## 3. Related Work

In the pursuit of creating smart buildings, many researchers have focused on the challenge of indoor localization. While GPS is widely regarded as a superior localization technology, it is not suitable for indoor positioning due to significant signal loss in such environments. As a result, alternative sensors and technologies like Bluetooth, ultrasound, and RFID have been explored. However, these options are often limited by factors such as energy consumption, cost, and bandwidth constraints. Conversely, WiFi technology has gained considerable attention recently, especially with the widespread use of smartphones in daily activities. This has made the development of WiFi-based systems more feasible and cost-effective, as they do not require specialized infrastructure. Among the most commonly used WiFi techniques are RSSI and time-based methods, which are discussed in this section concerning their relevance to our work. Leveraging Knowledge distillation in localization is also discussed in this section.

### 3.1. RSSI-Based Techniques

RSSI-based localization is a method used to determine the position of a device by measuring the strength of the wireless signals it receives. The basic idea is that the signal strength decreases as the distance from the transmitter, like a WiFi access point, increases. The accuracy of RSSI positioning techniques is often compromised by factors such as NLOS, fading, and data noise [42,43]. Despite these challenges, The simplicity of RSSI techniques has encouraged many researchers to explore ways to address and minimize these challenges. The RSSI-fingerprinting technique is one of the widely used and effective methods for overcoming these limitations [28,29,30]. This approach is a prime example of probabilistic localization techniques operating through two main stages: the offline phase and the online phase. In the offline phase, RSSI fingerprints of objects are gathered at designated reference points to build a database of these fingerprints. During the online phase, this database is utilized to estimate the positions of objects at new, unmeasured locations. Different features can be employed as system fingerprints.

Similarly, channel state information (CSI) techniques provide detailed insights into the signal conditions between two communicating nodes. Both RSSI and CSI localization methods are significantly influenced by fluctuations in WiFi node power, which is a common occurrence. Additionally, the performance of these techniques can be compromised by the variability among different WiFi devices. Although fingerprinting approaches are widely utilized for developing effective indoor localization systems, they face challenges such as sensitivity to signal interference, diffraction, and fading. Additionally, achieving efficient localization with fingerprinting requires a well-distributed and homogeneous placement of access points (APs).

### 3.2. Time-Based Techniques

Alternatively, time-based methods are frequently employed for indoor localization. These techniques calculate the positions of objects by measuring time and applying the known speed of the transmitted signal. Among the most widely used methods for this purpose are ToA [35,36], TDoA [37], and RTT [38]. The ToA technique measures the time it takes for a signal to reach the receiver station (timestamp). For precise time estimation, strict synchronization between both sides is crucial [35,36]. In contrast, TDoA techniques involve sending signals from three or more stations and measuring the differences in arrival times and signal propagation times to estimate the user’s location. While TDoA also requires time synchronization, it is needed only among the transmitters, unlike in ToA [37]. Both Time of Arrival (ToA) and Time Difference of Arrival (TDoA) are one-way measurement techniques. In contrast, Round-Trip Time (RTT) is a two-way measurement technique that determines the round-trip duration of a signal traveling from the transmitter to the receiver and back. A significant advantage of the RTT technique over ToA and TDoA is that it does not necessitate synchronization between the transmitter and receiver, as it relies on a single clock. However, traditional multilateration systems that utilize RTT measurements often face challenges with accuracy due to non-line-of-sight (NLOS) conditions and multipath effects [38].

Various WiFi-RTT techniques have been seen in the literature to minimize the impact of NLOS and multi-path effects. For instance, in [44], a real-time WiFi-RTT model was developed to address the errors caused by multipath and NLOS issues. In [45], the authors introduced a calibration model designed to correct transmitter RTT range offsets, thereby enhancing accuracy. Additionally, ref. [46] proposed a WiFi FTM geomagnetic positioning method to reduce the effects of NLOS, incorporating an enhanced mind evolutionary algorithm (EMEA) to improve localization accuracy. Meanwhile, ref. [47] developed a WiFi-RTT approach utilizing line-of-sight identification and range calibration. Other studies focus on detecting and classifying NLOS and multipath signals into low and high-quality categories using support vector machines [48]. To enhance positioning accuracy, ref. [9] proposes a hybrid RTT-RSSI technique that employs a new, straightforward multi-lateration model combining RTT and RSSI methods. This approach aims to improve localization precision, although it may not consistently achieve optimal results due to the potential signal attenuation caused by NLOS conditions. Additionally, ref. [17] introduces a hybrid RTT-RSSI fingerprinting localization method. However, the results indicate that this approach falls short of the desired accuracy because it does not account for the correlation between the different modalities.

### 3.3. Knowledge Distillation in Localization

Knowledge distillation is widely used in scenarios where maintaining model performance is important, but computational efficiency is also critical such as in real-time applications including smart home automation, emergency response systems, healthcare, etc. Localization plays a very important and crucial role in these applications. Leveraging knowledge distillation in localization shows its superiority as it runs efficiently on resource-constrained devices while still providing accurate location estimates. Thus, it becomes feasible to deploy high-performing localization models on smartphones or IoT devices. By utilizing knowledge distillation on localization, it is easier to scale across multiple devices or larger environments, allowing for the more widespread adoption of localization technology.

Several research efforts in the literature have explored the use of knowledge distillation in localization. For example, knowledge distillation is leveraged with RF-based localization to replace the sophisticated RF signal processing algorithm that is run based on visual signals with a simplified version designed to operate with limited computational resources [49]. Also, knowledge distillation has been employed to combine Bluetooth and ultrasound modalities in a lightweight model for people tracking in factories [50].

On the other hand, WiFi fingerprinting is a widely used technology for indoor localization due to the widespread availability of WiFi access points [51,52,53]. Knowledge distillation can make the WiFi more robust to environmental variations, such as changes in WiFi signal strength due to interference or obstacles. This adaptability enhances the model’s performance across different environments without needing extensive retraining.

In this paper, we introduce *DistilLoc*, a cross-knowledge distillation framework that transfers knowledge from an RTT-based teacher model to an RSSI-based student model. This teacher–student setup allows the highly accurate RTT models to inform more efficient RSSI models, making them better suited for real-time applications. The F-Net architecture is employed to capture complex relationships and dependencies between RSSI signals from multiple access points, enabling the student model to create a precise mapping from RSSI data to accurate location estimates.

## 4. System Overview

**Figure 2:**
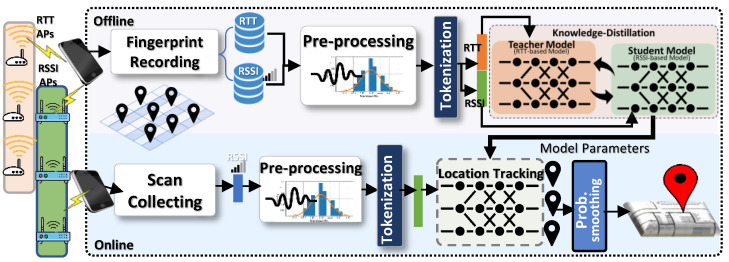
*DistilLoc* system architecture.

Figure 2 illustrates the architecture of *DistilLoc*, which operates in two primary stages: an offline calibration and training stage, followed by an online localization stage.

WiFi data are gathered during the offline phase from predefined reference points that are evenly distributed across the area of interest. The collected data typically consist of RTT and RSSI measurements from the overheard access points at each reference point, forming a comprehensive fingerprint map. This fingerprinting process is facilitated by the **Fingerprint Recorder** App, operating on a mobile device, and utilizing the Android RTT API [54] to detect both RTT and RSSI signals.

Once the fingerprint map is constructed, it is uploaded to the *DistilLoc* server for further processing. The **Pre-processor** module standardizes the data by constructing fixed-size vectors for both RSSI and RTT measurements, collected simultaneously from the APs. These vector pairs are then forwarded to the **Cross-Knowledge Distillation** module, where an F-Net-based teacher model is trained using both modalities. The teacher model extracts high-level, location-discriminative features, which are critical for precise location estimation. This high-quality teacher model is used to train a student localization model, which only relies on RSSI data during the runtime. By leveraging cross-modal knowledge distillation, the student model learns the complex signal relationships present in RTT data, allowing it to perform with high accuracy using only RSSI data during the online phase. The result of this stage is a trained student model, stored for future use.

In the online localization stage, real-time tracking begins as users carry their mobile devices in unknown locations. The devices continuously scan for APs, collect RSSI signals and forward these data to the *DistilLoc* server. The **Pre-processor** module constructs unified RSSI vectors from the collected signals, which are then passed to the trained student model. The student model, enriched by the knowledge distilled from the teacher model, predicts the most probable reference locations using the RSSI data. Despite not utilizing RTT during runtime, the student model provides high localization accuracy, effectively approximating the performance of RTT-based systems. The system ultimately outputs the user’s location in continuous spatial space, ensuring accurate, real-time localization.

## 5. The *DistilLoc* System

Figure 2 illustrates the various modules of the *DistilLoc* system. In this section, we will detail each module.

### 5.1. The Pre-Processor Module

The pre-processor module is tasked with mapping the RTT and RSSI measurements to pairs of fixed-length feature vectors. Each entry in a feature vector corresponds to a measurement from an AP, either RSSI or RTT. It is important to note that not all installed APs can be detected during every scan due to range limitations. Consequently, only a subset of the APs may be visible in any given scan, resulting in variable-length feature vectors. To address this issue, APs that are not detected during a specific scan are assigned an RTT value of $0.2\times 10^{-3}$ ms, which is equivalent to a distance of approximately 60 m. This value exceeds any RTT measurement for the APs within the scanning range. Similarly, an RSSI value of −100 dBm is assigned to any unheard AP, as this value is lower than all RSSI readings obtained from the reachable APs in the collected scans. Thus, a short RTT or low RSSI value is allocated to any AP that is far from the mobile device carried by the user. Moreover, it has been observed that when the mobile device is very close to an AP, the Android API [54] may report a negative distance. This can be attributed to the internal configuration and calibration of the WiFi cards, as well as the multipath compensation algorithms that process the measurements in firmware before they reach the driver. Additionally, RTT measurements can experience latency when used with rapidly moving mobile devices. The presence of such negative values (in the former case) or latency (in the latter case) typically results in a significant decline in the performance of traditional multilateration methods [20]. However, this does not adversely affect the performance of *DistilLoc*, as it employs a fingerprinting-based approach where such negative values or delays can serve as distinctive signatures for specific locations.

### 5.2. The Cross-Modal Knowledge Distillation Module

In this module, we present our methodology for improving the performance of an RSSI-based localization model (student) by distilling knowledge from a more accurate RTT-based model (teacher). We first introduce the structure of the teacher model, followed by a detailed explanation of the knowledge transfer mechanism to the student model.

#### 5.2.1. F-Net Architecture Details

**Figure 3:**
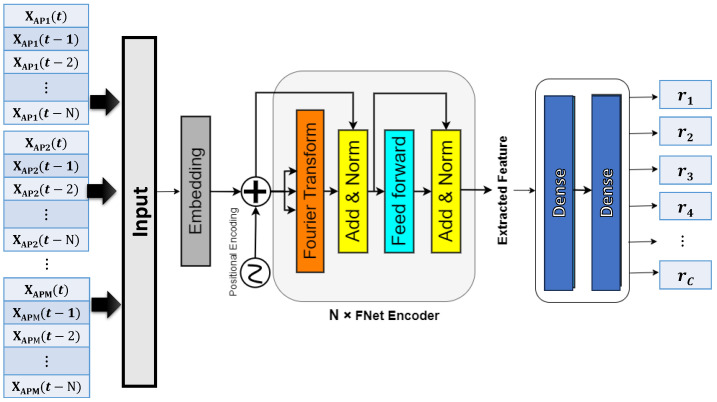
The network structure of the F-Net student model.

The F-Net architecture, depicted in Figure 3, serves as the backbone for both the teacher and student models, offering an efficient alternative to traditional transformers by replacing the self-attention mechanism with a Fast Fourier Transform (FFT)-based feature extraction process. This substitution significantly reduces computational complexity while preserving the model’s ability to capture global dependencies. Specifically, the complexity reduction from $O(N^{2})$ in self-attention to O(NlogN) in FFT makes F-Net highly efficient for tasks such as localization, where real-time inference and global correlation modeling are crucial.

**Figure 4:**
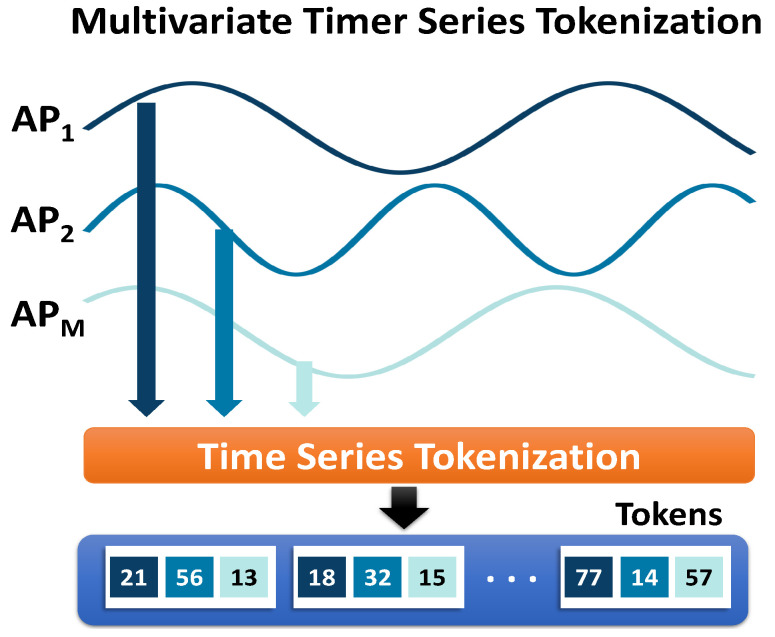
The Tokenization Process.

In *DistilLoc*’s teacher model, the input is typically a sequence of RTT measurements from multiple access points. To process this input effectively within the F-Net framework, it is necessary to tokenize the RTT values and prepare them in a format that the network can interpret. Tokenization (illustrated in Figure 4) is a critical step, as it enables the network to handle the sequential structure of the data while maintaining the relationships between the measurements from different APs over time. Unlike natural language processing, where tokens typically represent words or subwords, RTT tokenization requires careful consideration of the temporal and spatial relationships between measurements. Each RTT value $x_{i}$, where *i* represents the *i*-th access point, can be considered a token. However, since RTT data are collected over time, *DistilLoc* considers the input as multiple time windows, where each window $x_{t}=\left[x_{1,t},x_{2,t},...,x_{n,t}\right]$ serves as a token, where *t* denotes the time step and *n* is the number of APs. Tokenizing the RTT values into time-based (AP-based groups) allows the model to treat each group as an independent input unit, which is then processed through the network. This tokenization process is also essential for the model because it ensures that the RTT measurements are structured in a way that enables the network to capture both local interactions (e.g., between APs) and global dependencies (e.g., long-range correlations between APs over time).

Once the RTT input is tokenized, the tokens are embedded into a higher-dimensional space using a linear embedding layer. This is crucial for the model to learn complex interactions between the RTT readings. The embedding process is formalized as follows:

(11) $$X_{\mathrm{emb}}=XW_{\mathrm{emb}}+b_{\mathrm{emb}}$$

where $X\in R^{T\times n}$ represents the tokenized RTT measurements over *T* time steps, and *n* is the number of APs (or groups of APs). $W_{\mathrm{emb}}\in R^{n\times d_{\mathrm{emb}}}$ is the embedding weight matrix, and $b_{\mathrm{emb}}\in R^{d_{\mathrm{emb}}}$ is the bias term. The resulting $X_{\mathrm{emb}}$ is a high-dimensional representation of the tokenized RTT data, which facilitates the learning of complex signal interactions that are critical for accurate localization.

After embedding, the core operation of the F-Net applies the Fast Fourier Transform (FFT) to each row of $X_{\mathrm{emb}}$. The Fourier Transform is particularly well-suited for time-series and spatial data, as it enables the model to capture both periodic and global patterns in the signal, which are essential for accurately modeling the relationships between RTT readings from different APs. The Fourier Transform is defined as:

(12) $$F\left(X_{\mathrm{emb}}\right)=\mathrm{FFT}\left(X_{\mathrm{emb}}\right)$$

This transformation moves the data from the time (or spatial) domain to the frequency domain, where the periodic structures of the measurements can be analyzed more effectively. By capturing the frequency components of the RTT measurements, the FFT helps the model recognize correlations between signals that might not be evident in the raw data, such as periodic fluctuations caused by environmental dynamics.

The FFT produces complex-valued output, but for the sake of simplicity and computational efficiency, only the real part of the transformed data is used in subsequent layers:

(13) XFFT=Re(FFT(Xemb))

The real-valued FFT output retains the critical frequency-based information needed to model long-range dependencies between readings from different APs. This ability to capture global interactions efficiently is key to the success of F-Net in localization tasks, where signal interference, reflection, and multi-path effects can complicate the relationship between the user’s location and the observed RTT values.

Once the Fourier-transformed features are obtained, they are passed through a series of feed-forward neural networks, which introduce non-linearity and further refine the learned features. These layers are crucial for capturing non-linear relationships in the data and allow the model to build more salient representations of the input. The feed-forward operation is defined as follows:

(14) $$X_{\mathrm{FF}1}=\mathrm{ReLU}\left(X_{\mathrm{FFT}}W_{1}+b_{1}\right)$$

(15) $$X_{\mathrm{FF}2}=\mathrm{ReLU}\left(X_{\mathrm{FF}1}W_{2}+b_{2}\right)$$

where $W_{1},W_{2}\in R^{d_{\mathrm{emb}}\times d_{\mathrm{emb}}}$ are the weight matrices, and $b_{1},b_{2}\in R^{d_{\mathrm{emb}}}$ are the bias terms. The use of the ReLU activation function ensures that the network can model complex decision boundaries between different reference locations, which are necessary for precise localization.

The final output of the network is a set of logits z, which represent the predicted location classes. These logits are computed as follows:

(16) $$z=X_{\mathrm{FF}2}W_{\mathrm{cls}}+b_{\mathrm{cls}}$$

where $W_{\mathrm{cls}}\in R^{d_{\mathrm{emb}}\times C}$ is the classification weight matrix, *C* is the number of reference locations, and $b_{\mathrm{cls}}$ is the bias term. The logits are passed through a softmax function to produce the final location predictions, where the predicted probability of each reference location *i* is given by the following:

(17) $$P\left(r_{i}\right)=\frac{\exp(z_{i})}{\sum_{j}\exp\left(z_{j}\right)}$$

The model is trained using cross-entropy loss, which is well-suited for classification tasks:

(18) $$L=-\sum_{i=1}^{C}y_{i}\log\left({\hat{y}}_{i}\right)$$

where $y_{i}$ is the true label for the reference location, and ${\hat{y}}_{i}$ is the predicted probability for the corresponding location. The model is optimized using Adam with momentum, and the weight decay regularization technique is applied to prevent overfitting.

#### 5.2.2. Knowledge Transfer Mechanism

*DistilLoc* leverages Cross-Modal Knowledge Distillation (CMKD), integrating feature-stage and output-stage distillation within the F-Net architecture. RSSI-based models (e.g., WiDeep [51], and a Ranging-based system [20]) are limited by environmental factors such as signal multipath, interference, and obstacles, which degrade accuracy. On the other hand, RTT-based models (e.g., RRLoc [55], WiNar [17]) provide more reliable distance estimates but require specialized hardware that is not widely available. The *DistilLoc* approach addresses this gap by using a pre-trained RTT-based teacher model to improve the accuracy of an RSSI-based student model, allowing for the deployment of a highly accurate system using ubiquitous RSSI measurements.

Given input features $x_{RTT}\in R^{d_{t}}$ and $x_{RSSI}\in R^{d_{s}}$ corresponding to RTT and RSSI measurements, respectively, our objective is to train a student model $f_{s}\left(x_{RSSI};\theta_{s}\right)$ that can predict the location $y\in R^{2}$ with accuracy comparable to that of a teacher model $f_{t}\left(x_{RTT};\theta_{t}\right)$, where $\theta_{s}$, and $\theta_{t}$ are the learnable parameters for student and teacher models, respectively. The student model is trained using a combination of supervised learning, feature-stage distillation, output-stage distillation, and adversarial training.

Feature-stage distillation is a core component of our methodology, allowing the student model to learn hierarchical representations by aligning its intermediate feature maps with those of the teacher model. This process is critical because intermediate features often capture essential patterns that contribute to the final prediction, particularly in the localization tasks where spatial and contextual understanding are required.

Let $h_{t}^{l}\in R^{N\times d_{l}}$ and $h_{s}^{l}\in R^{N\times d_{l}}$ represent the feature maps at layer *l* of the teacher and student models, respectively, where *N* is the batch size and $d_{l}$ is the dimensionality of the feature map at layer *l*. The feature-stage distillation loss is defined as follows:

(19) $$L_{\mathrm{FD}}=\sum_{l=1}^{L}\frac{1}{N}\sum_{i=1}^{N}{∥h_{t,i}^{l}-h_{s,i}^{l}∥}^{2}$$

This loss encourages the student model to produce feature maps similar to those of the teacher, thereby inheriting the teacher’s ability to capture the complex relationships in RTT data. The novelty of this approach lies in its ability to transfer rich, intermediate representations across modalities, which is less explored in traditional distillation methods that focus primarily on output-stage distillation.

Output-stage distillation complements feature-stage distillation by aligning the final predictions of the teacher and student models. The logits (pre-softmax outputs) $z_{t}\in R^{C}$ and $z_{s}\in R^{C}$ from the teacher and student models, respectively, where *C* is the number of classes or output dimensions (i.e., reference locations), are transformed using a temperature-scaled softmax function:

(20) $$p_{t}=\mathrm{softmax}\left(\frac{z_{t}}{T}\right),\;p_{s}=\mathrm{softmax}\left(\frac{z_{s}}{T}\right)$$

The distillation loss $L_{\mathrm{KD}}$ is then computed as the Kullback–Leibler (KL) divergence between these softened distributions:

(21) $$L_{\mathrm{KD}}\left(p_{t},p_{s}\right)=T^{2}\cdot \mathrm{KL}\left(p_{t}∥p_{s}\right)=T^{2}\cdot \sum_{i}p_{t}\left[i\right]\log\left(\frac{p_{t}\left[i\right]}{p_{s}\left[i\right]}\right)$$

This output-stage distillation ensures that the student model mimics the final decision-making process of the teacher model. The introduction of temperature *T* in the softmax function serves to smooth the probability distribution, providing more informative gradients during training. This is crucial in order to allow the student model to learn from a teacher who operates on a different feature space. The temperature parameter T>1 controls the smoothness of the probability distributions, which helps in capturing the more nuanced relationships between classes that the student model can learn. The temperature scaling reduces the sharpness of the predictions, providing a richer set of target distributions that carry information beyond the hard labels, thus facilitating a more effective distillation process. The inclusion of the $T^{2}$ factor is crucial as it compensates for the temperature scaling in the gradient calculations, ensuring that the gradients remain meaningful and contribute effectively to the optimization process.

To ensure that the student model remains grounded in the actual location data, we incorporate a supervised loss based on the Mean Squared Error (MSE) between the predicted location ${\hat{y}}_{s}\in R^{2}$ and the true location $y\in R^{2}$:

(22) $$L_{\mathrm{MSE}}\left(y,{\hat{y}}_{s}\right)=\frac{1}{N}\sum_{i=1}^{N}{∥y_{i}-{\hat{y}}_{s,i}∥}^{2}$$

This supervised loss directly optimizes the student model’s predictions against the true labels, ensuring that the distillation process does not divert the model from the primary task of accurate localization.

To further enhance the robustness of the student model, we employ adversarial training, which is a novel addition to the CMKD framework. The goal of adversarial training is to make the student model’s features indistinguishable from those of the teacher model, even in the presence of adversarial perturbations. This method improves the student model’s resilience to variations in input data, such as noise or signal interference, which are common in indoor localization scenarios. In this setup, we introduce a generator network $G(h_{s}^{l};\theta_{G})$ that learns to produce feature representations for the student model that are indistinguishable from those of the teacher model. The discriminator network $D(h;\theta_{D})$ is trained to distinguish between the teacher’s feature maps $h_{t}^{l}$ and the student’s feature maps $h_{s}^{l}$. The adversarial loss is defined as follows:

(23) $$L_{\mathrm{adv}}=E_{x∼X}\left[\log D(h_{t}^{l})\right]+E_{x∼X}\left[\log(1-D\left(G\left(h_{s}^{l}\right)\right))\right]$$

The generator *G* aims to minimize the adversarial loss $L_{\mathrm{adv}}$ by generating features that the discriminator *D* cannot distinguish from the teacher’s features. Meanwhile, the discriminator seeks to maximize this loss to enhance its ability to differentiate between the generated and the teacher’s features. This adversarial framework is integrated into the training process to better align the student model’s feature space with the teacher’s, ultimately making the student model more resilient to adversarial conditions.

The final training objective for the student model integrates feature-stage distillation, output-stage distillation, supervised learning, and adversarial training:

(24) $$L_{\mathrm{total}}=\lambda_{\mathrm{FD}}\cdot L_{\mathrm{FD}}+\lambda_{\mathrm{KD}}\cdot L_{\mathrm{KD}}+\lambda_{\mathrm{MSE}}\cdot L_{\mathrm{MSE}}+\lambda_{\mathrm{adv}}\cdot L_{\mathrm{adv}}$$

where $\lambda_{\mathrm{FD}},\lambda_{\mathrm{KD}},\lambda_{\mathrm{MSE}},\lambda_{\mathrm{adv}}$ are hyperparameters controlling the weight of each loss component.

The combination of knowledge distillation with F-Net introduces several novel aspects. First, Feature-Stage Distillation Across Modalities goes beyond traditional distillation methods that focus mainly on output predictions by incorporating the distillation of intermediate features. These features are crucial for capturing spatial and contextual information necessary for accurate localization. Second, Adversarial Training for Cross-Modal Distillation is introduced within the CMKD framework, an area that has not been extensively explored in the literature. This component improves the student model’s ability to resist adversarial perturbations and noise, making it more robust in real-world scenarios. Lastly, F-Net integration enhances the efficiency and effectiveness of both the teacher and student models by using the F-Net architecture, which employs the Fourier Transform for feature extraction. This architecture is particularly well-suited for handling time-series data like RTT and RSSI, which is essential for localization tasks requiring the capture of periodic and global patterns in signal data.

The theoretical justification for CMKD stems from information theory. The KL divergence minimization in the distillation process reduces the information discrepancy between the teacher and student models. By aligning the output distributions, the student model effectively learns the decision boundaries and feature representations that the teacher model (trained on more accurate RTT data) has already learned. This process allows the student model to inherit the robustness and accuracy of the RTT model, even when only RSSI data are available at runtime.

### 5.3. Online Phase

The primary objective of the online phase is to estimate the user’s location in real-time using the signals received from overheard APs within the area of interest. This is achieved by processing the scanned AP information, extracting the corresponding feature vector through the trained F-Net student model, and feeding it into the localization head to obtain a location estimate at one of the pre-defined fingerprint points from the calibration phase. The reference point $r^{*}$ with the highest probability, given the input vector *x*, is selected as the estimated location. Formally, we aim to find the following:

(25) $$r^{*}=\underset{r}{\mathrm{argmax}}\left[P(r|x)\right]$$

However, a challenge arises in that the *Localization Model* predicts user locations only at discrete reference points. Even with a highly accurate model, the predictions are limited to these fixed points, potentially leading to a suboptimal user experience due to the spacing between these reference points. To address this issue, *DistilLoc* aims to track the user’s movement in continuous spatial space, allowing for localization at any point, even those not directly corresponding to the predefined reference points.

To achieve this, *DistilLoc* computes the center of mass of all reference points by applying a spatially weighted average. The weights for each point are determined by the softmax likelihood output from the classifier network, ensuring that the estimated location reflects a continuous space rather than being confined to discrete points. More formally, the estimated location $l_{x}$ and $l_{y}$ coordinates are calculated as follows:

(26) $$l_{x}=\frac{\sum_{i=1}^{n}P_{i}r_{ix}}{\sum_{i=1}^{n}P_{i}},\;l_{y}=\frac{\sum_{i=1}^{n}P_{i}r_{iy}}{\sum_{i=1}^{n}P_{i}}$$

where $r_{ix}$ and $r_{iy}$ are the spatial coordinates of reference point *i*, and $P_{i}$ represents the softmax likelihood for that point. This approach enables *DistilLoc* to provide a more seamless and accurate user experience by tracking the user in a continuous spatial domain, rather than limiting location estimates to the predefined reference points.

## 6. Evaluation

This section first outlines the data collection setup and the tools employed. Next, we demonstrate the system’s performance by varying different system parameters. Finally, we compare *DistilLoc*’s performance to that of state-of-the-art techniques.

### 6.1. Collection Setup and Tools

To analyze and evaluate the performance of the *DistilLoc* system, we deployed it in two realistic indoor testbeds. Table 1 summarizes the characteristics of these two testbeds.

**Figure 5:**
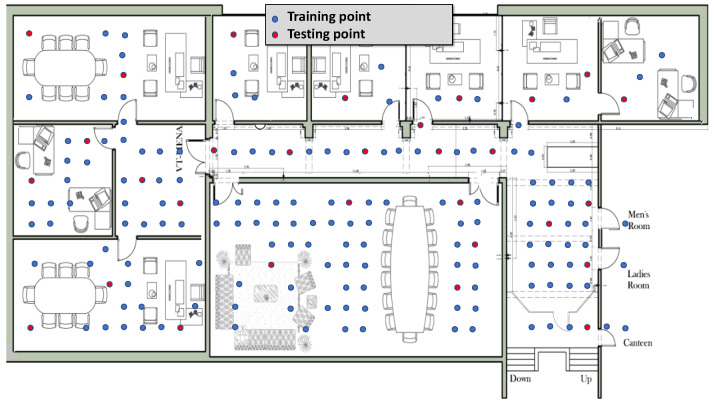
The Lab testbed layout. Blue circles represent training points, while red circles indicate testing points.

The first testbed, referred to as “Lab”, is a full floor in our university campus, covering an area of 629 m^2^. It includes nine rooms of various sizes and a long corridor, as illustrated in Figure 5.

**Figure 6:**
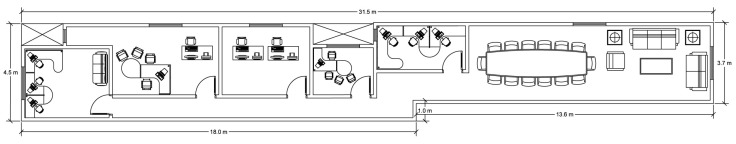
The Office testbed layout.

The second testbed, labeled “Office”, is depicted in Figure 6. It is an administrative building with an area of 141 m^2^, featuring a large meeting room, a long corridor, and five additional rooms. Seven Google WiFi APs are evenly distributed in both testbeds to ensure comprehensive coverage of the entire area of interest.

The area of interest in both testbeds is uniformly divided into various reference points, spaced one meter apart from each other (the impact of altering the spacing between reference points will be evaluated later in this section). The Lab testbed features 143 distinct reference locations, whereas the Office testbed comprises 76 locations. Each reference location is guaranteed to be covered by at least one Google WiFi access point (AP).

Data were gathered using an Android application installed on various Android phones, including the Google Pixel XL and Pixel 2 XL. The application continuously searches for nearby APs. To ensure accurate ground-truth profiling, the employed data collector application operates synchronously on all mobile devices, with one device designated to manage ground-truth collection for all devices. The user inputs the coordinates of their current location (ground truth) and initiates the data collection process. At each reference location, a minimum of 100 samples is gathered within a 3-min timeframe for training purposes. Independent hold-out test sets were collected, comprising 21 and 30 locations (distinct from the training points) in the Office and Lab testbeds, respectively. This data collection occurred over several days during working hours to account for the temporal variation of indoor signals.

### 6.2. Ablation Study

In this section, we analyze the impact of various system parameters on the performance of *DistilLoc*. These parameters include reference point spacing, AP density, temperature for distillation, modality, and distillation type, The goal is to understand how these parameters and modalities influence localization accuracy and system robustness, particularly under different environmental conditions. For clarity of presentation, the results in the following subsections focus on the Lab testbed. However, in Section 6.3.1, we compare the system’s performance across both the Lab and Office testbeds to provide a more comprehensive evaluation.

Table 2 summarizes the default values of the system parameters that are used throughout the evaluation section. These values serve as a baseline, with variations applied to individual parameters to assess their effect on the system’s overall performance.

#### 6.2.1. Effect of Temperature Parameter

**Figure 7:**
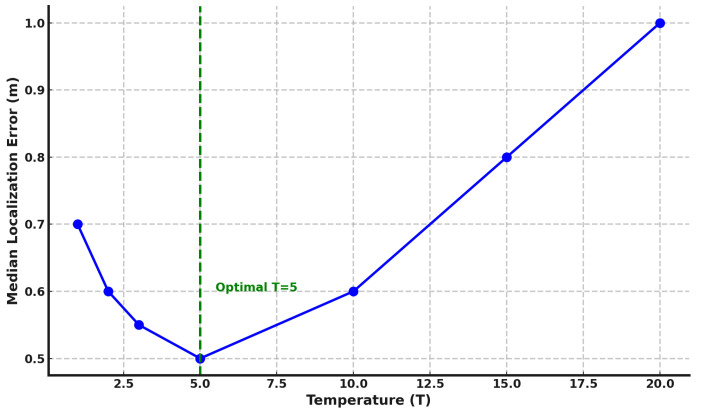
Effect of temperature parameter on median localization error during the distillation process.

In this section, we study the effect of different temperature values used in the knowledge distillation process on the performance of the *DistilLoc* system. The temperature parameter plays a critical role in the softmax function used during distillation, controlling the smoothness of the output probability distribution from the teacher model. Figure 7 shows the relationship between temperature (*T*) and the median localization error. As shown in the figure, the system’s performance is highly sensitive to the choice of temperature. At lower temperatures (e.g., T=1), the system achieves a relatively high error of 0.7 m, indicating that the output distributions from the teacher model are not sufficiently softened, resulting in the student model struggling to effectively learn from the teacher. As the temperature increases, we observe a steady improvement in performance, with the error dropping to 0.5 m at T=5. This indicates that at this temperature, the softmax function smooths the teacher’s output distribution just enough to provide meaningful gradients, allowing the student model to learn a more generalized and effective representation of the localization task. Beyond T=5, however, the performance starts to degrade. At T=10, the median localization error increases to 0.6 m, and at T=20, the error further worsens, reaching 1.0 m. This decline suggests that excessively high temperatures overly soften the teacher’s output, causing the student model to lose the sharp distinctions between classes. As a result, the model becomes less effective in distinguishing the correct location. The results indicate that T=5 is the optimal temperature for the distillation process in the *DistilLoc* system. At this temperature, the student model effectively balances learning from the teacher’s softened output while maintaining accurate class distinctions.

The optimal temperature may vary with environmental conditions, particularly in spaces with unique characteristics like high interference, reflective surfaces, or significant multipath effects. For instance, environments with complex layouts or dense obstructions might benefit from a slightly adjusted temperature to improve the robustness of feature transfer. While our experiments confirmed T=5 as effective in two different environments, in other environments, fine-tuning the temperature could further optimize model performance.

#### 6.2.2. Effect of Reducing the Density of APs

In this section, we analyze the impact of reducing the number of RTT-capable and RSSI-capable access points (APs) on the performance of the *DistilLoc* system. The experiments simulate the effect of removing APs, and the corresponding percentage reductions.

**Figure 8:**
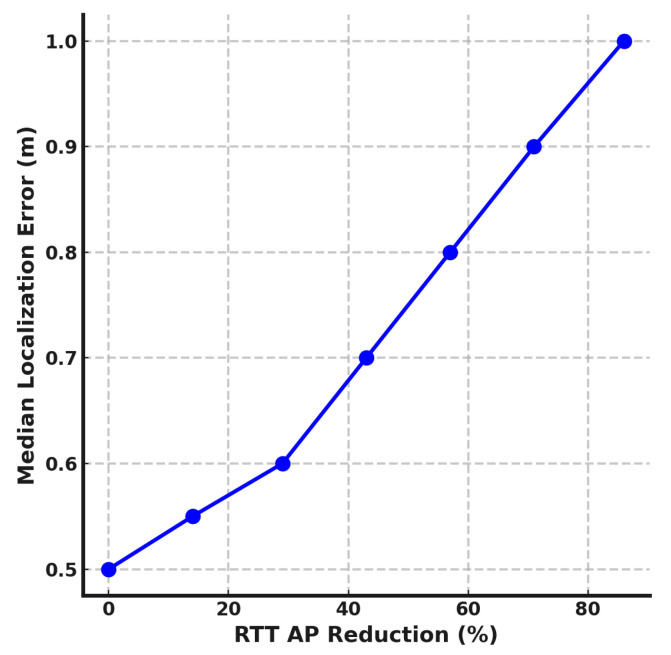
Impact of reducing the density of RTT-capable APs on median localization error in the offline phase.

Figure 8 shows how the median localization error is affected by reducing the number of RTT-capable APs during the offline phase. Starting with seven RTT-capable APs, no reduction (0%), the system achieves a median localization error of 0.5 m. As the number of RTT-capable APs decreases, we observe a steady increase in localization error. A reduction of one RTT-capable AP (approximately 14%) increases the error to 0.55 m. Further reduction, removing six of the seven APs (86%), leads to a significant deterioration in performance, with the error rising to 1.0 m. This trend demonstrates the sensitivity of the *DistilLoc* system to the availability of RTT-capable APs. RTT measurements offer high precision in distance estimation, and reducing the number of such APs compromises the system’s ability to maintain accurate location estimates. The results suggest that at least three RTT-capable APs are required in the offline phase to keep the localization error below 0.7 m. In environments where high accuracy is critical, maintaining a sufficient density of RTT-capable APs is essential.

**Figure 9:**
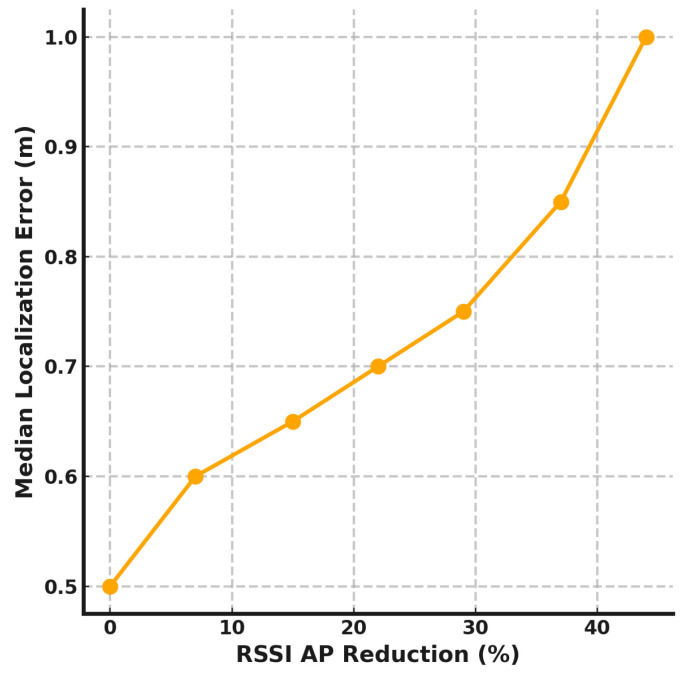
Impact of reducing the density of RSSI-capable APs on median localization error in the online phase.

Figure 9 presents the effect of reducing the density of RSSI-capable APs during the online phase. Starting with 136 APs, the system initially achieves a median error of 0.5 m. Removing 10 APs (approximately a 7% reduction) causes the error to increase slightly to 0.6 m. A reduction of 60 APs (44% reduction) leads to an error of 1.0 m, showing a gradual performance decline. The results indicate that the system is more resilient to RSSI-capable AP reduction compared to RTT-capable AP reduction. This highlights the advantage of the proposed *DistilLoc* system that enhances the performance of RSSI-based localization leveraging RTT-capable APs only during the offline phase. While RTT-capable APs provide higher precision and more immediate localization benefits, the system can tolerate a higher reduction in RSSI-capable APs, making it a practical solution for dynamic environments.

#### 6.2.3. Effect of Fingerprint Points Density

**Figure 10:**
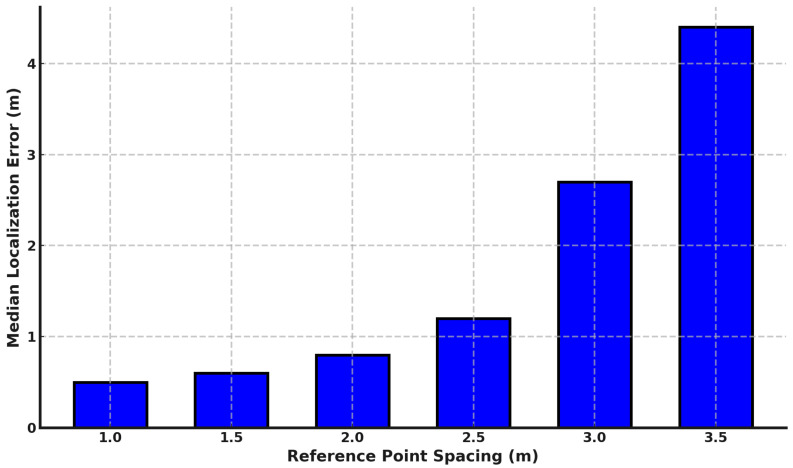
Impact of increasing reference point spacing on median localization error.

In this section, we examine the effect of increasing the spacing between fingerprint points on the performance of *DistilLoc*. As shown in Figure 10, larger spacing between reference points leads to a gradual degradation in localization accuracy. However, compared to traditional RSSI-based systems [51], *DistilLoc* demonstrates greater robustness to reductions in fingerprint density. Specifically, the system’s median localization error only increases by 30 cm when the spacing is doubled from 1 m to 2 m. This modest performance loss can be attributed to *DistilLoc*’s cross-modal knowledge distillation approach, where the teacher model, trained on more reliable RTT measurements, helps the student model (which relies on noisier RSSI data) generalize better even with fewer reference points. The distillation process ensures that the student model can extract more informative and robust features from the available fingerprints, thus reducing the dependency on the density of the fingerprint grid. This robustness is further reinforced by *DistilLoc*’s feature extraction process, which uses deep learning models capable of capturing the nonlinear relationships between RTT and RSSI signals. However, beyond a spacing of 2.5 m, the system begins to show a more significant drop in accuracy. At 3 m, the median error reaches 2.7 m, and at 3.5 m, it rises sharply to 4.4 m. This sharp increase can be explained by the reduced granularity in the spatial representation of the environment. At larger spacings, the system has fewer reference points to accurately capture the subtle variations in signal strength, making it harder to precisely estimate the user’s location. Moreover, the system’s ability to handle multipath effects and environmental clutter is diminished as fewer fingerprints are available to account for these variations.

#### 6.2.4. Performance Comparison of Different Modalities

**Figure 11:**
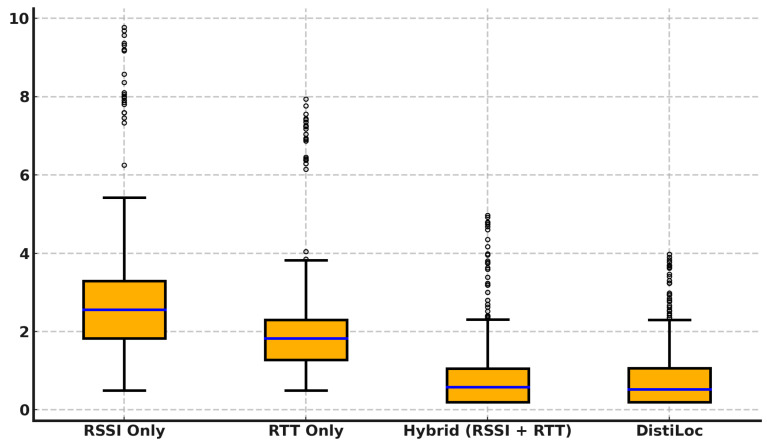
Performance of different modalities.

Figure 11 presents the localization error for four techniques: RSSI Only, RTT Only, Hybrid (RSSI + RTT), and *DistilLoc*. The results clearly demonstrate that *DistilLoc* outperforms the other methods, achieving the lowest median error with minimal outliers. The RSSI-only method shows the highest median error (around 2.5 m) due to the inherent variability in signal strength, resulting in a wide error distribution and numerous outliers. RTT-only, on the other hand, achieves better accuracy with a median error of around 0.5 m, leveraging more stable time-based measurements. The Hybrid approach, combining both RSSI and RTT, further reduces the error (approximately 0.3 m), demonstrating the benefits of fusing both modalities. *DistilLoc* performs comparably to the hybrid method but with a slightly narrower error distribution, indicating its stable performance. This improvement can be attributed to the knowledge distillation process, which enables *DistilLoc* to maintain high accuracy using only RSSI during runtime. The significantly reduced number of outliers in *DistilLoc* highlights its robustness and consistency in various environments.

#### 6.2.5. Distillation Type Impact on Localization Accuracy

To evaluate the impact of different distillation techniques on the performance of the proposed DistiLoc system, we conducted a series of experiments comparing four distinct approaches: no distillation, feature-stage distillation, output-stage distillation, and a combined approach that leverages both feature-stage and output-stage distillation.

**Figure 12:**
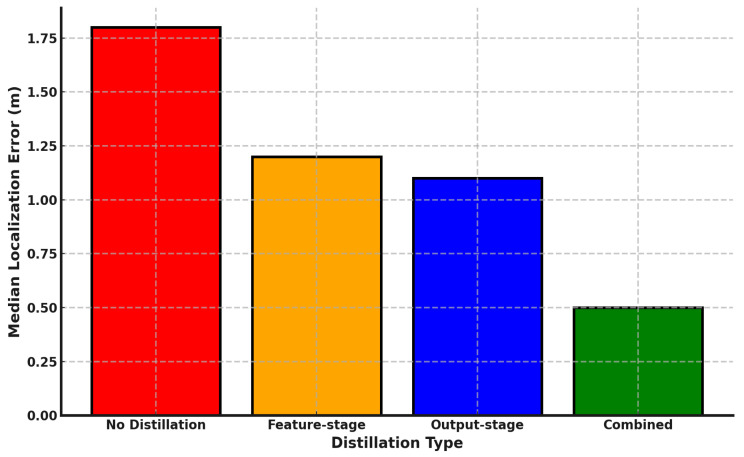
Distillation type impact in the Office testbed.

Figure 12 shows that the DistiLoc system, without any distillation technique, served as the baseline, yielding a median localization error of 1.8 m. This relatively high error underscores the challenges faced by the system when relying solely on raw signal data without the refinement provided by distillation. The introduction of output-stage distillation resulted in an improvement, reducing the median error to 1.2 m. This reduction can be attributed to the model’s ability to align its output predictions more closely with those of the teacher model, which has been trained on more accurate RTT data. By focusing on the final output probabilities, the student model benefits from the well-established decision boundaries of the teacher, leading to enhanced localization accuracy. Feature-stage distillation further improved the performance, bringing the median localization error down to 1.1 m. This approach enables the student model to learn richer hierarchical representations by aligning its intermediate feature maps with those of the teacher model. By capturing more nuanced patterns within the signal data, feature-stage distillation enhances the model’s robustness and ability to generalize across varying environments. The most substantial improvement was observed with the combined approach of all distillation, achieving a median localization error of just 0.5 m. The combined approach leverages the strengths of both distillation methods, providing the student model with comprehensive guidance throughout the entire learning process. The alignment of both intermediate features and final predictions ensures that the student model not only captures detailed patterns within the data but also makes decisions that closely mirror those of the more accurate teacher model. These findings not only validate the efficacy of knowledge distillation in the DistiLoc system but also highlight the potential of combining multiple distillation techniques to transfer knowledge from more accurate but less accessible models (e.g., RTT-based models) to those that rely on more ubiquitous but noisier data sources like RSSI.

### 6.3. Comparative Evaluation

This section compares the performance of *DistilLoc* with four WiFi-based localization systems: RRLoc [55], WiNar [17], WiDeep [51], and a Ranging-based system [20].

RRLoc [55] fuses RTT and RSSI with the deep network version of canonical correlation analysis to obtain embeddings, which are then used for location classification. It necessitates the presence of both RTT and RSSI during both the offline and online phases. WiNar [17] utilizes a deterministic method that aligns captured RTT measurements with a pre-recorded fingerprint map to estimate the user’s location while incorporating RSSI values to adjust the weight of the estimated locations. WiDeep [51] constructs an RSSI-based localization system using a deep denoising autoencoder neural network. The system described in [20] (referred to as Ranging) utilizes a multilateration approach based on RTT for indoor localization while detecting NLOS conditions. All techniques were assessed using the same dataset to ensure a fair comparison.

#### 6.3.1. Localization Accuracy

**Figure 13:**
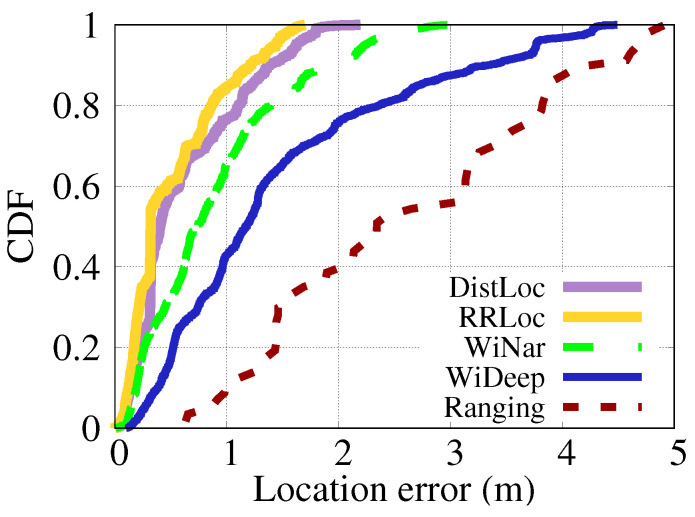
Comparison of CDFs of different systems in the office testbed.

**Figure 14:**
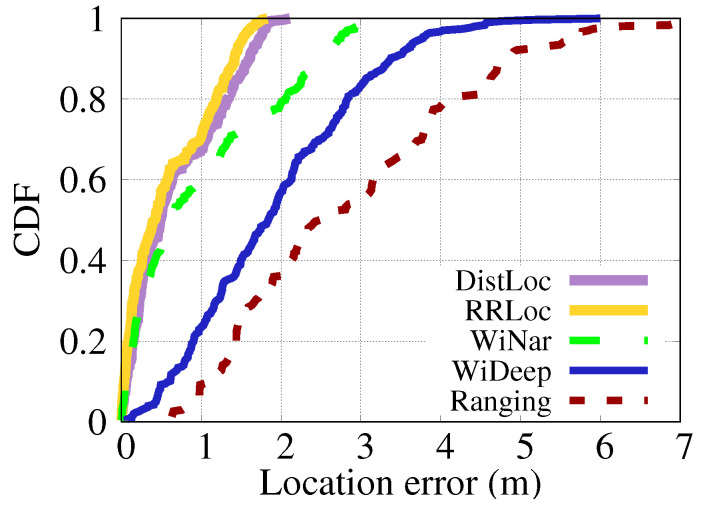
Comparison of CDFs of different systems in the Lab testbed.

Figure 13 and Figure 14 present the CDF of localization errors for various techniques across two different testbeds. The results demonstrate that *DistilLoc* achieves substantial improvements in localization accuracy compared to other systems. Figure 13 which represents the smaller testbed, *DistilLoc* outperforms WiNar [17], WiDeep [51], and Ranging-based systems [20] by 48.98%, 138.78%, and 377.55%, respectively, in terms of median localization error. Additionally, *DistilLoc* achieves a performance comparable to RRLoc [55], with only a marginal difference of 17 cm. This improvement is significant, as *DistilLoc* operates without requiring RTT during runtime, unlike RRLoc, which relies on both RTT and RSSI for accurate positioning. The key advantage of *DistilLoc* stems from its use of knowledge distillation, allowing it to learn from RTT data during the training phase while solely relying on RSSI measurements during operation. This innovative approach ensures similar performance to RTT-based systems, even in environments where RTT data are unavailable.

In Figure 14, which focuses on the larger Lab testbed, the results show that *DistilLoc* continues to exhibit superior performance. The median localization error is improved by 5.00% over RRLoc [55], 52.50% over WiNar [17], 360.00% over WiDeep [51], and an impressive 527.50% over the Ranging-based systems. These findings underline the scalability and robustness of *DistilLoc* when applied in larger and more complex environments. This remarkable improvement in both testbeds can be attributed to *DistilLoc*’s ability to transfer knowledge from RTT-based models (teacher) to RSSI-based models (student) via cross-modal knowledge distillation. By distilling the more accurate RTT signal characteristics into the RSSI-based system, *DistilLoc* significantly enhances its ability to mitigate signal noise, interference, and multipath effects—common challenges in indoor localization. Additionally, the F-Net architecture’s ability to capture complex signal relationships further enhances *DistilLoc*’s capacity to generate accurate localization estimates even in cluttered environments.

In summary, as shown in Table 3 and Table 4, *DistilLoc* outperforms all other systems across all percentiles in both testbeds. This improvement is primarily due to its advanced knowledge distillation mechanism, enabling it to emulate the high precision of RTT-based systems while maintaining the simplicity and accessibility of RSSI-only systems.

#### 6.3.2. Time per Location Estimate

**Figure 15:**
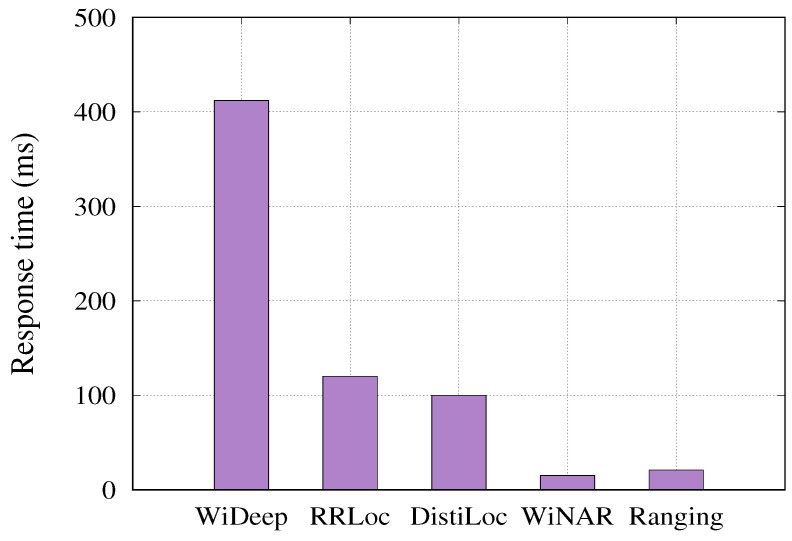
Comparison of run time of the different systems.

We evaluated the running time of the different systems using a Lenovo Thinkpad X1 laptop equipped with a 2.2 GHz Intel i7-8750H processor, 64 GB of RAM, and an Nvidia GTX1050Ti 4GB GPU. The results are presented in Figure 15. As expected, *DistilLoc*, RRLoc [55], and WiDeep [51], being deep learning-based systems, require more time for each location estimate compared to traditional methods. This is because these systems must pass the input through multiple layers of the network to produce an estimate, whereas deterministic methods like WiNar [17] and Ranging-based techniques [20] rely on simpler calculations, leading to shorter response times. However, *DistilLoc* demonstrates a significant advantage over WiDeep [51]. WiDeep assigns a separate neural network to each reference point, which greatly increases its computational overhead. In contrast, *DistilLoc* uses a more efficient model architecture that generalizes across reference points, resulting in faster location estimates. Additionally, *DistilLoc* shows a slightly faster response time compared to RRLoc [55], which relies on a complex network of multiple subnetworks (one for each modality), adding extra layers of computation. Despite these differences in running time, all systems, including *DistilLoc*, are capable of providing real-time tracking. The current response times are well within acceptable limits for most applications and could be further optimized through parallelization if necessary. This makes *DistilLoc* not only a highly accurate system but also a practical choice for real-time localization applications, striking a balance between computational efficiency and localization precision.

#### 6.3.3. Device Heterogeneity

**Figure 16:**
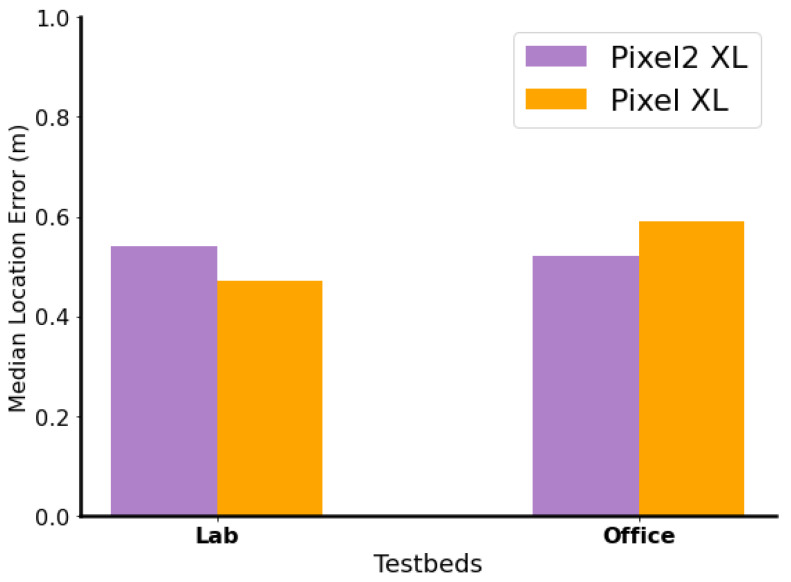
Effect of varying the testing device on *DistilLoc* performance in the two testbeds.

In this section, we evaluate the robustness of *DistilLoc* to device heterogeneity, where training data are collected on one device and testing is performed on a different device. Specifically, we examine the performance when training is conducted on a Pixel 2 XL and testing is performed on a Pixel XL, and vice versa, across both the Lab and Office testbeds. Figure 16 demonstrates that *DistilLoc* consistently performs well, regardless of which device is used for testing, with only a slight performance advantage observed when the Pixel 2 XL is used. This consistency is significant, as different mobile devices exhibit hardware variations that directly impact RSSI measurements. These variations include differences in form factors, antenna locations, chipset designs, and signal processing capabilities. These hardware-related factors often introduce offsets in the measured RSSI, as seen in prior studies [56], leading to performance variability in traditional RSSI-based systems. However, *DistilLoc* successfully mitigates these challenges through the use of cross-modal knowledge distillation, which incorporates both RSSI and RTT data during training. While RSSI is prone to hardware-induced variations, RTT measurements are largely unaffected by device-specific factors due to their time-based nature [38]. By leveraging the stability of RTT signals during the training phase, *DistilLoc* reduces the impact of device heterogeneity on localization accuracy. The system learns a more robust feature representation that generalizes well across different devices, thus minimizing the influence of hardware-specific RSSI variations during testing. This approach ensures that *DistilLoc* maintains high localization accuracy, even in heterogeneous device scenarios.

While our proposed system demonstrates significant improvements in localization accuracy, several practical challenges must be considered for large-scale deployment. The main obstacles include:

- Infrastructure and Hardware Costs: Achieving high localization accuracy with RTT-capable APs requires a level of infrastructure upgrade, as these APs are not yet widely available and may be cost-prohibitive in large installations. Many existing buildings rely on standard WiFi APs, which lack RTT capabilities, meaning a full transition to RTT-capable APs could necessitate substantial investments in both new hardware and installation.
- Compatibility with Legacy Devices: For effective localization, user devices need to be RTT-compatible. Although RTT support is growing, especially in newer smartphones, many legacy devices lack this capability, limiting the system’s reach and effectiveness if used in environments with diverse device types.
- Environmental Constraints: Real-world environments present unique challenges, such as uneven AP distribution, signal interference, and varying construction materials that affect signal propagation. In such cases, selective deployment strategies or hybrid models may be needed to balance coverage, accuracy, and cost. Adjusting the density and location of APs to accommodate specific building layouts or high-traffic areas can mitigate some of these issues but requires careful planning.
- Maintenance and Scalability: The cost and complexity of maintaining a high-accuracy localization infrastructure can be substantial, especially in large facilities with frequently changing layouts or usage patterns. Scalability is also a concern, as additional APs may be required in new areas, necessitating further costs and calibration efforts.

To address these obstacles, we suggest a phased approach to deployment, beginning with hybrid solutions that combine existing RSSI-based APs with strategically placed RTT-capable APs in high-priority areas. This approach minimizes initial costs while ensuring a foundation for gradual infrastructure enhancement. Additionally, knowledge distillation allows the student model to leverage RTT-based accuracy even when RTT data are unavailable at runtime, enabling the system to maintain higher accuracy in mixed AP environments.

## 7. Conclusions

Indoor localization remains a challenging problem due to factors such as environmental noise, multipath fading, and signal interference, particularly those relying on WiFi RTT. These challenges hinder the development of efficient, real-time localization systems, especially for resource-constrained devices. To address these limitations, we have introduced *DistilLoc*, a novel cross-knowledge distillation framework that transfers the precision of RTT-based models to more efficient RSSI-based models. By employing a teacher–student approach, where the teacher model uses WiFi RTT and the student model relies on WiFi RSSI, *DistilLoc* enables the transfer of high accuracy to less computationally demanding models, making it suitable for real-time applications. The incorporation of the FNet architecture further enhances the system by capturing the complex relationships among RSSI signals from multiple access points, enabling the student model to generate precise location estimates with reduced computational overhead. The experimental results, conducted across two indoor testbeds, demonstrate that *DistilLoc* outperforms traditional RSSI fingerprinting and ranging-based multilateration techniques, achieving high accuracy even across heterogeneous devices.

Beyond its technical achievements, this work also contributes to the broader goals of Sustainable Development Goals SDG 9 and SDG 11. By improving the efficiency and scalability of indoor localization systems, *DistilLoc* supports the development of smart, resource-efficient technologies that are critical for building sustainable, connected urban environments. As such, *DistilLoc* represents not only an advancement in localization technology but also a step forward in the creation of smarter, more sustainable cities.

## Figures and Tables

**Table 1:** Summary of the testbeds used to evaluate the *DistilLoc* system.

Criteria	Lab Testbed	Office Testbed
Area (m^2^)	17 × 37	4.5 × 31.5
No. of training points	143	76
No. of testing points	30	21
Spacing of seed points (m)	1	1
Building Material	Brick	Brick and Wood
No. of APs	7	7
Total fingerprinting time (hrs:mins)	∼08:39	∼04:51
Training time (hrs:mins)	∼01:05	∼0:43

**Table 2:** Default parameters values used in the evaluation.

Parameter	Range	Default
Learning rate	0.0001–0.2	0.001
Batch size	1-Dataset size	128
Number of layers	1–30	3
Early Stopping Patience (epochs)	1–10	40
Number of samples per reference point	20–100	100
Number of epochs	Automatic by Early stopping	
Used devices	Google Pixel XL, Google Pixel 2XL	
Number of users	3	
Update rate (scan/sec)	2	

**Table 3:** The localization error percentiles in the Office testbed.

Technique	Average	25th Percentile	50th Percentile	75th Percentile	Maximum
***DistilLoc***	**0.68 m**	**0.21 m**	**0.49 m**	**1.21 m**	**2.11 m**
*RRLoc*	0.51 m (25.0%)	0.19 m (9.5%)	0.32 m (34.7%)	0.79 m (34.7%)	1.70 m (19.4%)
*WiNar* [17]	0.89 m (−30.9%)	0.34 m (−61.9%)	0.73 m (−48.9%)	1.20 m (0.83%)	2.99 m (−41.7%)
*WiDeep* [51]	1.46 m (−114.7%)	0.58 m (−176.2%)	1.17 m (−138.8%)	1.97 m (−62.8%)	4.49 m (−112.8%)
*Ranging* [20]	2.59 m (−280.9%)	1.44 m (−585.7%)	2.34 m (−377.5%)	3.68 m (−204.1%)	4.92 m (−133.2%)

**Table 4:** The localization error percentiles in the Lab testbed.

Technique	Average	25th Percentile	50th Percentile	75th Percentile	Maximum
***DistilLoc***	**0.629 m**	**0.28 m**	**0.40 m**	**0.94 m**	**2.19 m**
*RRLoc*	0.59 m (6.2%)	0.12 m (57.1%)	0.42 m (−5.0%)	1.08 m (−14.9%)	1.83 m (16.4%)
*WiNar* [17]	0.99 m (−57.4%)	0.19 m (32.1%)	0.61 m (−52.5%)	1.77 m (−88.3%)	3.00 m (−37.0%)
*WiDeep* [51]	1.92 m (−205.2%)	1.06 m (−278.5%)	1.84 m (−360.0%)	2.69 m (−186.1%)	6.00 m (−173.9%)
*Ranging* [20]	2.86 m (−354.7%)	1.46 m (−421.4%)	2.51 m (−527.5%)	3.85 m (−309.5%)	7.38 m (−237.0%)

## Data Availability

Data are contained within the article.
